# Les hémodialysés HVC sont-ils vraiment des patients difficiles à traiter?

**DOI:** 10.11604/pamj.2015.22.373.8353

**Published:** 2015-12-16

**Authors:** Khadija Krati, Hind Cherquaoui, Sofia Oubaha, Zouhour Samlani

**Affiliations:** 1 Service de Gastroentérologie, Centre Hospitalier Universitaire Mohamed VI de Marrakech, Marrakech, Maroc; 2 Laboratoire de Physiologie, Faculté de Médecine et de Pharmacie de Marrakech, Marrakech, Maroc

**Keywords:** Hépatite virale C, hémodialyse, réponse virologique soutenue, facteurs prédictifs de bonne réponse thérapeutique, viral hepatitis C, hemodialysis, sustained virologic response, Predictors of good treatment response

## Abstract

L'hépatite C demeure la principale infection virale chez l'hémodialysé, dont la prise en charge thérapeutique ainsi que la gestion de ses effets secondaires restent difficiles. Il s'agit d'une étude rétrospective portant sur tous les patients atteints d'hépatite C chronique ayant une insuffisance rénale chronique sous hémodialyse, suivis au service de gastroentérologie au CHU Mohamed VI de Marrakech sur une période de Janvier 2004 à Décembre 2014. Sur un total de 355 cas d'hépatite virale C, 13 patients étaient hémodialysés (3,66%). Dix patients ont été traités, soit 76,94% des cas. Le traitement n’était pas indiqué chez 2 patients ayant une fibrose minime sans cytolyse. Il était contre-indiqué chez une patiente multitarée. Deux malades ont eu une réponse virologique rapide et 5 une réponse virologique précoce. Le taux de réponse virologique soutenue était de 40%, 30% des patients étaient non répondeurs. Le traitement fut arrêté chez 2 patientes pour effets secondaires sévères. Un seul patient a été candidat à une transplantation rénale. En analyse multivariée, la réponse virologique soutenue a été significativement associée à certains facteurs prédictifs de bonne réponse thérapeutique: Age jeune ≤40 ans (P=0,0057), fibrose minime F1-F2 (P=0,03), génotype non 1 (P=0,0064), charge virale préthérapeutique <800000 UI/ml (P=0,013), et l'absence d'arrêt thérapeutique (P=0,028). La gestion efficace des effets secondaires du traitement de HVC permet d'obtenir chez l'hémodialysé un taux de réponse virologique soutenue avoisinant celui de la population générale.

## Introduction

Les infections virales notamment celles dues au virus de l'hépatite C (VHC), de l'hépatite B sont fréquentes chez les malades soumis à une hémodialyse chronique. L'hépatite virale C (HVC) demeure la principale infection virale chez les malades hémodialysés. La gravité de cette infection réside dans son risque élevé d’évolution vers la chronicité et du développement d'une cirrhose ou d'un hépatocarcinome. Malgré une forte prévalence de l'infection HVC chez les hémodialysés; les études existantes sur le traitement de cette population considérée « difficile à traiter » sont rares. L'objectif principal de ce travail est de décrire notre expérience dans la prise en charge des patients atteints d'hépatite virale C hémodialysés.

## Méthodes

Il s'agit d'une étude rétrospective sur dossiers de patients HVC suivis en consultation de Janvier 2004 à Décembre 2014 au service de gastroentérologie au CHU (centre hospitalier universitaire) Mohamed VI de Marrakech. Ont été inclus tous les patients atteints d'hépatite virale C chronique ayant une insuffisance rénale chronique sous hémodialyse, naïfs de toute thérapeutique. Les patients éligibles au traitement ont tous bénéficié d'un bilan préthérapeutique, qui une fois normal, ont débuté un traitement par interféron pégylé (INF peg) en monothérapie ou en association à de faibles doses de ribavirine (RBV). La durée du traitement est établie en fonction du génotype du virus: elle est de 48 semaines chez les patients ayant un VHC de génotype 1 ou 4 et de 24 semaines chez les patients ayant un VHC de génotype 2. La charge virale du VHC (ARN viral) et le taux des transaminases étaient les principaux points de suivi. La tolérance clinique et biologique a été également évaluée. Les variables quantitatives sont exprimées en médianes et en extrêmes et les valeurs qualitatives sont exprimées en pourcentage. La comparaison des variables qualitatives est réalisée à l'aide du test exact de Fisher en utilisant le logiciel SPSS 10.0. Le seuil de signification était P < 0,05.

## Résultats

**Figure 1:**
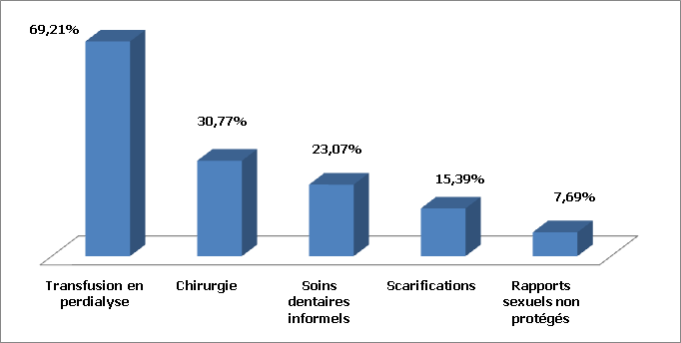
Principaux facteurs de risque de transmission virale hépatitique

**Figure 2:**
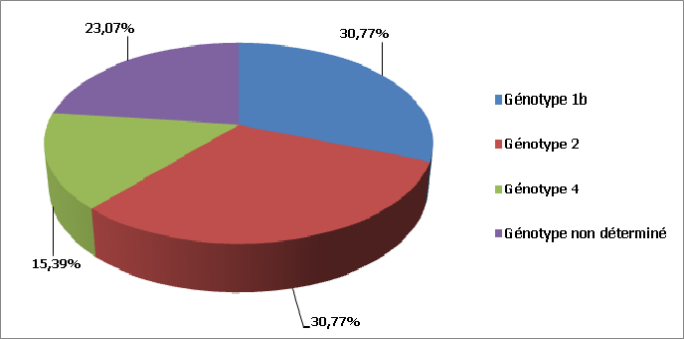
Répartition des patients selon le génotype

Sur un total de 355 cas d'hépatite virale C, 13 patients étaient des insuffisants rénaux chroniques sous hémodialyse, soit un taux de 3,66%. L’âge moyen de nos patients était de 48 ans avec des extrêmes allant de 24 ans à 79 ans. Une prédominance féminine a été notée dans 53,86% des patients avec un sexe ratio F/H de 1,16. Quatre patients avaient comme antécédents une hypertension artérielle (HTA) chronique sous traitement (soit 30,77% des cas), une patiente était suivie pour une insuffisance cardiaque avec arythmie cardiaque par fibrillation auriculaire (ACFA), une autre patiente était suivie pour dysthyroidie, un patient avait une hépatite virale B guérie et un autre patient était diabétique type I. Les principaux facteurs de risque de transmission virale hépatitique chez nos patients étaient la transfusion en perdialyse chez 9 patients (69,21%), la chirurgie chez 4 patients (30,77%), des soins dentaires informels chez 3 patients (23,07%), les scarifications chez 2 patients (15,39%) et les rapports sexuels non protégés chez un seul patient (Figure 1). Les patients étaient dialysés depuis en moyenne 10 ans avec des extrêmes allant de 1 à 27 ans, à raison de 2 séances par semaine (chez 76,94% des cas). Seuls 3 malades avaient une néphropathie initiale connue expliquant l'hémodialyse: le premier avait une néphropathie diabétique, le deuxième avait un rein hypoplasique et une troisième patiente avait eu une néphrectomie gauche d'origine non déterminée. L'infection virale a été découverte en moyenne 4,5 ans après le début de l'hémodialyse chez 7 patients (53,86%), le plus souvent lors d'un bilan étiologique d'asthénie chez 5 patients (38,47%), ou d'ictère cholestatique chez 2 patients (15,39%). Seuls 6 patients (46,14%) ont bénéficié d'un dépistage systématique d'HVC avant l'hémodialyse ayant posé le diagnostic. La charge virale médiane pré-thérapeutique était de 4351611,84 UI/ml. Huit patients (61,54%) avaient une charge virale inférieure à 800000 UI/ml. Huit patients (61,54%) avaient, au Fibroscan, une fibrose modérée à sévère (F2-F4). Les transaminases étaient ≥ 2 fois la normale chez 8 patients (61,54%). Dans cette série, le génotype (G) 1b a été retrouvé dans 4 cas (30,77%), le génotype 2 dans 4 cas (30,77%), le génotype 4 dans 2 cas et 3 cas étaient non précisés (Figure 2). Dix patients ont été traités sur les 13 patients HVC hémodialysés inclus dans cette étude, soit 76,94% des cas. Le traitement n’était pas indiqué chez 2 patients ayant une fibrose minime F0-F1 sans cytolyse hépatique. Il était contre-indiqué chez une troisième patiente qui était multitarée (âge avancé (79 ans), HTA, insuffisance cardiaque avec ACFA). La dose médiane de IFN Pegα2a/α2b était de 135 et 80 µg/semaine respectivement, après la séance de dialyse. La ribavirine était dosée à 200 mg/j (1 seul comprimé). Six patients ont bénéficié d'une bithérapie pégylée (60% des cas), alors que 4 (40% des cas) ont été mis sous interféron pégylé seul. Des effets secondaires classiques du traitement ont été notés chez la majorité des patients (80%), telle l'anémie. Cette dernière a fait arrêter rapidement la ribavirine de façon définitive chez 4 patients (40%) et transitoire chez 2 patients (20%), ceci malgré le recours à l’érythropoïétine (EPO) (un recours limité chez seulement 60% des patients). Des transfusions sanguines ont été mises en cours de traitement chez 2 patients. Trois patients ont présenté une anémie sévère (Hémoglobine <8,5 g/dl) ayant nécessité un arrêt transitoire de la bithérapie pégylée. Deux malades ont eu une réponse virologique rapide (20%), 5 une réponse virologique précoce (50%) et 4 une réponse virologique en fin de traitement (40%). Les transaminases s’étaient normalisées chez 70% des patients. Au final, le taux de réponse virologique soutenue (RVS) était de 40% (G1b: 25%, G2: 75%), 3 patients (30%) étaient non répondeurs (G4: 66,6%, G1b: 33,4%), dont un patient G1b présentait une mutation IL 28 B C/T. Le traitement fut arrêté transitoirement chez 2 patientes: la première avait présenté une hyperplasie de l'endomètre révélée par des méno-métrorragies abondantes, la deuxième avait présenté une hypothyroidie profonde. Une patiente a été perdue de vue. Un seul patient a été candidat à une transplantation rénale (sur une liste d'attente). En analyse multivariée, la réponse virologique soutenue a été significativement associée à certains facteurs prédictifs de bonne réponse thérapeutique: Age jeune ≤40 ans (P=0,0057), fibrose minime F1-F2 (P=0,03), génotype non 1 (P=0,0064), charge virale préthérapeutique <800000 UI/ml (P=0,013), et l'absence d'arrêt thérapeutique (P=0,028) (Tableau 1). Par ailleurs, la RVS n’était pas significativement associée au sexe (P=1).

**Tableau 1:** Facteurs prédictifs de réponse virale soutenue en étude multivariée

Facteurs prédictifs de bonne réponse thérapeutique	Significativité statistique
Age jeune ≤ 40 ans	P=0,0057*
Fibrose minime F1-F2	P=0,03*
Génotype non 1	P=0,0064*
Charge virale préthérapeutique <800000 UI/ml	P=0,013*
Absence d'arrêt thérapeutique	P=0,028*

* Test exact de Fisher

## Discussion

Les modes de contamination sanguine (transfusions) et nosocomiale du VHC (dans les centres d´hémodialyse) [1] expliquent la fréquence de l´infection par le VHC chez les malades dialysés. L´existence d´une infection par le VHC dans ces populations a un impact important sur l´indication de la transplantation rénale chez ces malades en raison de l´augmentation de la mortalité après la greffe [2]. Il est donc indispensable de dépister de façon systématique l´hépatite C et d´évaluer sa sévérité histologique dans ce contexte afin d’éviter la diffusion de l'infection et de traiter précocement les patients. L´infection par le VHC est fréquente chez les malades insuffisants rénaux hémodialysés avec une prévalence variant entre 10 et 65% en fonction des zones géographiques [3]. Elle est significativement associée à la durée de l´hémodialyse et au nombre de produits sanguins transfusés [4]. Elle a diminué de façon importante avec le développement des mesures d´hémovigilance [5] et les précautions universelles d´hygiène. Les modes de contamination à l´intérieur des centres restent mal connus, mais l´efficacité des mesures d´hygiène et la prévalence plus élevée de l´infection à VHC en hémodialyse qu´en dialyse péritonéale ou en dialyse à domicile suggèrent plus une transmission interhumaine, sans doute manuportée par le personnel, qu´une transmission par l´équipement de dialyse lui-même [6]. Les anomalies biologiques (augmentation de l´activité des aminotransférases) semblent moins fréquentes (environ 33%) chez les malades insuffisants rénaux que dans la population générale des malades atteints d´infection chronique par le VHC (environ 75%) [7]. Dans notre étude, les transaminases étaient ≥2 fois la normale chez 8 patients (61,54%). Sur le plan virologique, la capacité de synthèse d´anticorps est diminuée chez les malades insuffisants rénaux, expliquant un pourcentage relativement élevé des faux négatifs des tests ELISA ou RIBA [4]. La séroconversion est en outre souvent retardée, bien après le délai habituel de 10 semaines observé dans la population générale. Les tests de troisième génération, plus sensibles, ont nettement diminué le risque de faux négatifs. De la même façon, des cas de séroréversion, définie par la disparition des anticorps anti-VHC détectables dans le sang malgré une virémie persistante, ont été décrits chez les dialysés comme chez les transplantés rénaux [8]. La PCR est donc le test diagnostique le plus efficace chez les malades insuffisants rénaux. Elle permet d´affirmer la présence d´une réplication virale. La biologie moléculaire permet aussi d´identifier le génotype et de quantifier la charge virale [9]. Chez les malades insuffisants rénaux, comme chez les malades contaminés par transfusion sanguine, le génotype le plus fréquent est le génotype 1b en Europe et au Japon [10]. Ce génotype était présent chez 4 patients (30,77%) dans notre travail.

L´impact de l´infection par le VHC sur la morbidité et la mortalité des hémodialysés a été insuffisamment apprécié, mais plusieurs études ont suggéré une diminution de la survie des dialysés infectés par le VHC [11]. Dans une étude histologique, une cirrhose était présente dans 11% des cas [12]. Enfin, il a été suggéré que l´espérance de vie des dialysés infectés par le VHC était meilleure après transplantation rénale qu´en cas de poursuite de la dialyse [13]. L´aggravation potentielle de l´hépatopathie après transplantation rénale et la contre-indication à l´utilisation de l´interféron chez le malade greffé rénal posent la question du traitement antiviral chez les candidats à une transplantation rénale, quelles que soient les lésions histologiques afin de tenter d´obtenir une éradication virale avant la greffe. Le traitement de l´infection par le VHC chez l'hémodialysé comporte 2 volets: préventif et curatif. La prévalence de l´infection à VHC a significativement diminué depuis la mise en place de différentes mesures préventives [14]. Néanmoins, malgré l´efficacité du dépistage systématique des dons du sang et de l´utilisation large d´érythropoïétine, la contamination par le VHC persiste avec une incidence actuelle de 0 à 1,4% par an selon les centres, principalement par transmission nosocomiale [15]. En pratique, le diagnostic d´une infection virale C chez un sujet hémodialysé indique la réalisation d´une biopsie hépatique. Chez les dialysés qui ne sont pas candidats à la transplantation rénale, le traitement est indiqué, comme pour la population générale, en cas d´hépatopathie significative et en l´absence de comorbidités sévères, notamment cardiovasculaires. Dans notre étude, le traitement était contre-indiqué chez une patiente ayant des tares cardiovasculaires. En l´absence de traitement, nous conseillons une échographie annuelle et une évaluation de la fibrose hépatique (par des méthodes invasives ou non invasives) tous les 3 à 5 ans pour ne pas méconnaître une éventuelle indication thérapeutique. Chez les malades en attente de transplantation, l´évaluation histologique a pour but principal de rechercher une cirrhose. En effet, la cirrhose contre-indique une transplantation rénale seule en raison de son pronostic péjoratif après transplantation rénale. Dans ce cas, une double transplantation foie-rein doit être envisagée [16]. Si le patient dialysé cirrhotique est traité et qu´il existe une réponse virologique durable, une biopsie hépatique de contrôle est réalisée. En effet, nous avons observé des cas de réversibilité de la cirrhose chez certains patients traités, ceux-ci ne relevant alors que d´une seule transplantation rénale [17].

Le but du traitement antiviral chez le patient avant transplantation rénale est l´éradication virale quel que soit le stade histologique pour améliorer le pronostic après greffe [18]. Au cours des dernières années, le traitement de l´hépatite virale chronique C a considérablement progressé avec l´apparition de nouvelles molécules antivirales ciblant spécifiquement le virus: les anti-protéases. Les bithérapies pégylées “classiques” associant la ribavirine et l´interféron pégylé ont évolué vers des trithérapies associant une antiprotéase. Beaucoup de nouveaux antiviraux apparaissent depuis peu et d´autres font actuellement l'objet d'essais cliniques totalement nouveaux réalisant des trithérapies voire des quadrithérapies sans interféron ou sans ribavirine. La mise à disposition de cette nouvelle génération d'antiviraux d'action directe annonce une révolution dans les traitements des personnes porteuses d'infection chronique par le virus de l'hépatite C: mieux tolérés, plus efficaces, avec des données disponibles aujourd'hui qui permettent d'espérer la guérison virologique de plus de 90% des malades après une cure de 12 ou 24 semaines seulement. Actuellement, plusieurs options sont disponibles pour traiter les patients hémodialysés [19]: un traitement par Sofosbuvir + Simeprevir, un traitement par Sofosbuvir + Daclatasvir, un traitement par Sofosbuvir + Ledipasvir, un traitement par Paritaprevir/ritonavir + Ombitasvir ± Dasabuvir. Chez les patients de génotype 1 ayant une clairance de créatinine <30 ml/min/1,73m^2^, le traitement par Grazoprevir + Elbasvir pendant 12 semaines sera le schéma thérapeutique recommandé. Chez les patients hémodialysés, les schémas thérapeutiques sans ribavirine sont à privilégier. Une posologie de 200 mg/j ou tous les 2 jours ou 200 mg 3 fois par semaine après la dialyse est recommandée. En effet, pour des raisons pharmacocinétiques, l´accumulation de métabolites de la ribavirine dans les érythrocytes au cours du traitement peut être responsable d´anémies sévères et prolongées malgré l´augmentation des doses d´érythropoïétine. Les principales études chez les patients dialysés sont résumées dans le Tableau 2 [20–24]. Même si les séries sont peu nombreuses et concernent peu de patients, les taux de réponse virologique soutenue varient entre 28 et 99%, le notre était à 40%, ce qui correspond bien aux données de la littérature.

**Tableau 2:** Principales études de traitement de l'HVC chez les patients dialysés.

Auteurs	Traitement	Nombre de patients /G	Durée du traitement	RVS (%)	Effets secondaires
Notre étude	INF Peg +/-RBV	4/G1b 4/G2 2/G4	48 semaines 24 semaines 48 semaines	40	Anémie chez 80% des patients: arrêt de RBV, EPO, transfusion-Hypothyroidie
Carriero et al [20]	INF Peg + RBV	12/G1	48 semaines	28	2 décès suite à des maladies cardiovasculaires, anémie
Mousa et al [21]	INF + RBV	20/G1	24 semaines	66	
Van Leusen et al [22]	INF Peg + RBV	5/G1 2/G2	48 semaines 24 semaines	80	Réduction de dose de Peg, transfusions itératives
Czul et al [23]	Sofosbuvir (200mg/j) + Simeprevir (150mg/j)	19/G1	12 à 24 semaines	88	
Roth et al [24]	Grazoprevir + Elbasvir	116/G1	12 semaines	99	

## Conclusion

En pratique, l´HVC doit être dépistée chez l'hémodialysé par la recherche d´anticorps anti-VHC et, au moindre doute, par une PCR VHC. Son traitement doit être principalement préventif basé sur le respect des règles d'hygiène universelles, mais aussi curatif basé sur les antiviraux directs.
